# Expression of Concern: A new method for ethical and efficient evidence generation for off-label medication use in oncology (a case study in glioblastoma)

**DOI:** 10.3389/fphar.2023.1264737

**Published:** 2023-07-31

**Authors:** Frontiers Editorial Office

With this notice, Frontiers states its awareness of concerns regarding the METRICS study described in this article. This expression of concern has been posted while Frontiers investigates the allegations. The situation will be updated as soon as the investigation is complete.

**UPDATE:** This article has now been retracted. Please find the full retraction here: https://www.frontiersin.org/journals/pharmacology/articles/10.3389/fphar.2026.2021924

